# Modelling of Backscattering off Filaments Using the Code IPF-FD3D for the Interpretation of Doppler Backscattering Data

**DOI:** 10.3390/s22239441

**Published:** 2022-12-02

**Authors:** Alexander Yashin, Natalia Teplova, Georgiy Zadvitskiy, Anna Ponomarenko

**Affiliations:** 1 Plasma Physics Department, Peter the Great Saint Petersburg Polytechnic University, 195251 Saint Petersburg, Russia; 2 Plasma Research Laboratory, Ioffe Institute, 195251 Saint Petersburg, Russia; 3 Max-Planck-Institut für Plasmaphysik, 85748 Garching, Germany

**Keywords:** filaments, tokamak, plasma diagnostics, Doppler backscattering, simulations

## Abstract

Filaments or blobs are well known to strongly contribute to particle and energy losses both in L- and H-mode, making them an important plasma characteristic to investigate. They are plasma structures narrowly localized across a magnetic field and stretched along magnetic field lines. In toroidal devices, their development is observed to take place in the peripheral plasma. Filament characteristics have been studied extensively over the years using various diagnostic techniques. One such diagnostic is the Doppler backscattering (DBS) method employed at the spherical tokamak Globus-M/M2. It has been observed that the DBS signal reacts to the backscattering from filaments. However, the DBS data have proven difficult to analyze, which is why modelling was undertaken using the code IPF-FD3D to understand what kind of information can be extrapolated from the signals. A circular filament was thoroughly investigated in slab geometry with a variety of characteristics studied. Apart from that, the motion of the filaments in the poloidal and radial directions was analyzed. Additionally, other shapes of filaments were presented in this work. Modelling for the real geometry of the Globus-M/M2 tokamak was performed.

## 1. Introduction

Fluxes of power and particles to plasma-facing components are a concerning phenomenon for magnetic confinement devices (tokamaks and stellarators), particularly for future fusion devices, such as ITER [1], as these fluxes can result in damage to the machine that can significantly affect the core plasma performance [2]. A variety of factors play a role in energy and particle transport with it resulting from a competition between sources of parallel and perpendicular losses. Filaments or blobs (filament-like plasma perturbations) are well known to strongly contribute to particle and energy losses both in L- and H-mode regimes [3], making them an important plasma characteristic to investigate. They are plasma structures that are narrowly localized across a magnetic field while being stretched out for up to several meters along magnetic field lines. In toroidal devices, their development is observed to take place in the peripheral plasma, close to the last closed flux surface (LCFS) or separatrix [4,5,6].

Filament characteristics have been studied extensively over the years using various diagnostic techniques [7]. One such diagnostic technique (unconventional though it may be for this purpose) is the Doppler backscattering (DBS) method [8], which was initially employed at the spherical tokamak Globus-M [9,10,11,12,13] and later the ASDEX Upgrade tokamak [14] for the purpose of studying filaments. It has been observed that the DBS signal reacts to backscattering from filaments. The reaction manifests itself as a burst of coherent fluctuations (CF) of the measured IQ (In-phase and Quadrature) signals. However, the measurements collected using DBS have proven difficult to analyze, which is why modelling needs to be undertaken to understand what kind of information can indeed be extrapolated from the signals. The first results of such modelling were obtained in the work in [15] describing the reaction of the DBS signal to a filament with set parameters such as its shape, direction of its motion and trajectory; it contains data regarding the possible reaction of the DBS diagnostics to changes in these parameters. While the results allowed for some understanding of the filaments in Globus-M, it should be noted that the modeling presented in the work in [15] possessed a number of erroneous conclusions, which are discussed in this paper.

This work built on the recent observations of filaments that have illuminated that there is much work still to be done in understanding the types of filaments observed in Globus-M2 [16]. For this reason, modelling was undertaken using the code IPF-FD3D [17] in slab geometry. One aspect that needs to be more thoroughly investigated is the circular filament interpretation. While some simulation data were analyzed so as to understand the experimentally detected filaments (this, however, highlighted that this proposed model cannot be applicable for all types of filaments observed), there are other characteristics that need to be modelled when it comes to circular filaments. Apart from that, the motion of filaments has been experimentally investigated in detail [18], which provided an insight into the fact that the filaments can move in different directions (even having rather complex trajectories). This makes it an important aspect to be modelled for DBS diagnostics, as the data collected can provide characteristics such as the direction of the moving filaments and their velocity. Additionally, experimental measurements [19] have raised several questions that include the possibility of the development of filament structures of different shapes, which are presented in this work.

The paper is structured as follows. The next section contains the conditions of the modelling that was undertaken. After that, the circular filament was studied in detail, with the impact of its various parameters as well as its different motion on the DBS signals presented. Then, the strip filament model was investigated. After that, data for different types of stretched filaments are presented. All these findings are then compared to data obtained for a different density profile. Finally, the DBS signals for the real Globus-M2 tokamak geometry are discussed.

## 2. Materials and Methods

Two-dimensional full-wave simulations were conducted with the finite-difference time-domain (FDTD) code IPF-FD3D [17] in slab geometry. The FDTD scheme is a time-steeping iterative scheme for solving Maxwell’s equations on a regular grid. The FDTD method is used in the IPF-FD3D code to simulate electromagnetic wave propagation in cold plasma, which is described by the following system of equations:

(1) $$\frac{\partial J}{\partial t}=\varepsilon_{0}\omega_{pe}^{2}E-\omega_{ce}J\times \hat{B_{0}}$$

(2) $$\frac{\partial E}{\partial t}=\frac{1}{\varepsilon_{0}}\nabla \times H-\frac{1}{\varepsilon_{0}}J$$

(3) $$\frac{\partial H}{\partial t}=-\frac{1}{\mu_{0}}\nabla \times E$$

where J is the plasma current, the plasma density is described by plasma frequency $\omega_{pe}^{2}$, the strength of the background magnetic field is given by frequency $\omega_{ce}$, and its direction is given by $\hat{B_{0}}$. The electric E and magnetic H fields of the electromagnetic wave are described by Maxwell’s Equations (2) and (3). In the code, these partial differential equations are translated into finite difference equations, and then the fields E and H and the current J are calculated consecutively in a leap-frogging time scheme. The IPF-FD3D code implements a one-, two-, and three-dimensional solver of Equations (1)–(3) on a cartesian grid. In this paper, the 2D solver was used. In addition to the equation-solving engine, the code incorporates such elements as numeric antennas, in phase/quadrature (I/Q) detectors for optical mixing and phase measurement, and ideally absorbing boundary conditions at the edge of the computation grid. The code is mainly applied to Doppler reflectometry and the investigation of microwave components; however, it can be used for other purposes as well. The main weaknesses of the FDTD method are, first, the inflexible grid geometry that does not allow for adaptation to the geometry of the modeled structures, and second, the need to calculate many time steps until a stationary state is reached. While the former is not relevant to the modeling of smoothly varying plasma densities, the latter, in combination with the need for gathering statistics on turbulent processes, leads to substantial use of computational resources, as a high number of realizations is computed. Therefore, the supercomputer of the Supercomputer Center of the Peter the Great St. Petersburg Polytechnic University was used for the calculations.

**Figure 1:**
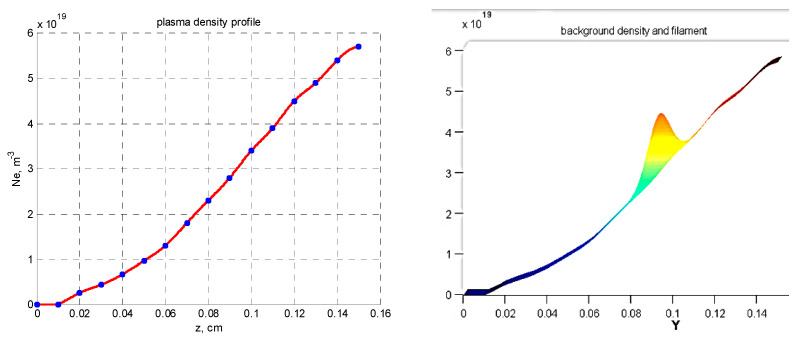
(**left**) Experimental electron density profile for Globus-M2, (**right**) experimental electron density profile for Globus-M2 with introduced filament distribution.

The simulation setup is organized as follows. For a given series of conditions set for the filament, the simulations obtain the I and Q signals of the quadrature detector, which can be compared to the experimental measurements obtained using DBS. For the purpose of understanding a variety of data collected, real experimental scenarios were examined. The electron density profile (referred to as the high-density profile) used in the simulations was taken from Thomson measurements and is presented on the left in Figure 1. The experimental plasma density profile used for the computations was close to linear, with values reaching n_e_ = 5.8 × 10^−19^ m^−3^ at y = 0.15 m (shot no. 36569 at Globus-M).

The approach to modeling the filaments involves introducing a filament density distribution on top of the chosen density profile. This is demonstrated on the right in Figure 1, which contains the resulting density profile of the combined background and filament density. The model of the filament was relatively simple with, a Gaussian cross-section that allowed freedom in choosing the shape, size, amplitude, and position of the filament.

The parameters that corresponded to the conditions of the performed modelling are presented in Table 1. These include the size of the box, step size, coordinates of the equatorial plane and plasma edge, position of the antenna, its tilt angle, and others.

**Figure 2:**
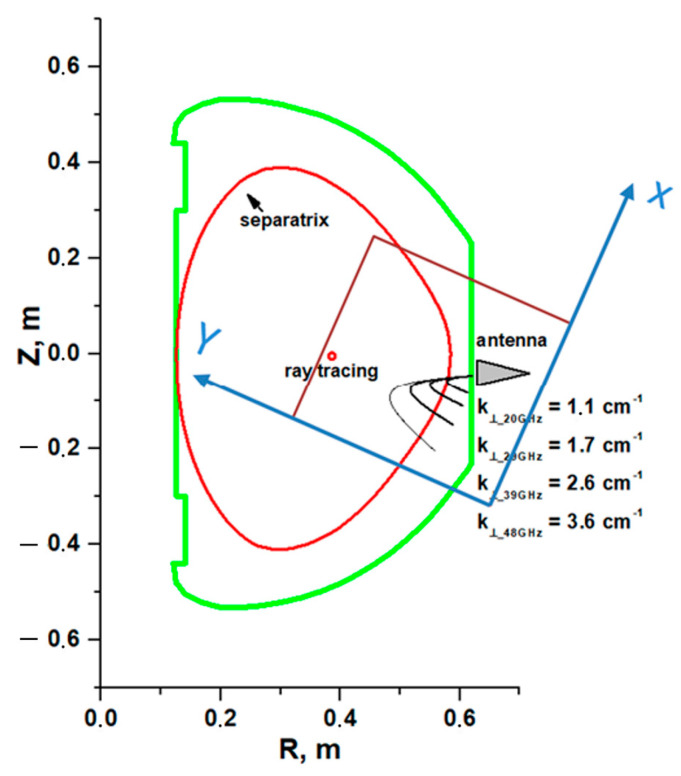
Poloidal cross-section of the Globus-M2 tokamak with performed ray tracing.

Figure 2 schematically represents the position and size of the box where the calculations were performed in regard to the Globus-M/M2 poloidal cross-section. The antenna is demonstrated with its position in the equatorial plane by the grey triangle, and the series of black lines are the calculated probing beam trajectories using a ray-tracing code for the Globus-M/M2 geometry. The box was placed to entirely include the DBS system, i.e., the antenna and the probing beam trajectories. The parameters of the DBS antenna of the installed system were as follows: antenna tilt angle α = 13°, antenna horn mouth 5.5 cm, and a Gaussian beam with a flat wave front in the antenna mouth. The coordinate system of the box is also demonstrated, with the *X* axis corresponding to the poloidal direction (parallel to the magnetic field lines), and the *Y* axis to the radial direction in the tokamak.

**Figure 3:**
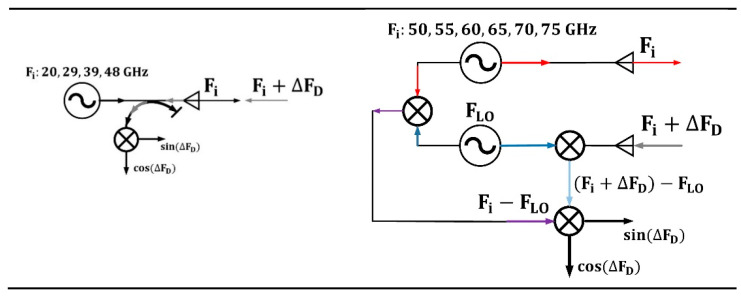
Schematics of DBS systems in Globus-M2.

Computations were performed for a range of probing frequencies, i.e., 20, 29, 39, 48, 50, 55, 60, 65, 70, and 75 GHz, in O-mode, which corresponded to the one in the installed systems in the Globus-M2 tokamak. Their schematics are presented in Figure 3. The system on the left is the low-frequency system, which uses homodyne detection, and the one on the right is the high-frequency system that employs heterodyne detection. The systems are described in detail in the works in [20,21].

One of the goals of this work was to investigate different circular filaments, which meant that a level of flexibility in their parameters was necessary. Different values of their size, amplitude (percentage of density at the cutoff of the probing wave), position of the filament, and a range of probing frequencies (a variety of cut-off radii) were necessary to increase the database of circular filaments in comparison to the results in paper [15]. All the parameters of the circular filaments are presented in Table 2, where all the values in italics represent the additional data obtained. All these various parameters were analyzed to observe the transition from linear to nonlinear scattering, as the purely linear model was unable to explain all experimental data.

Apart from changes to the filament itself, its motion was investigated. As recent research suggests, depending on the location where the filament is developed in, it can move in different directions, with rather complex trajectories also being possible [17]. Thus, it was important to include this aspect into the modelling, with the poloidal and radial directions being added to the calculations (Table 3). The code also allowed for complex trajectories (meaning not in just one direction) to be implemented. In the case of purely poloidal or purely radial motion, the position of the filament was changed with a change in the probing frequency. The filament motion was simulated by independent snapshots with a spatial step of 1 mm. If a time interval was assigned equal to this step, the velocity of the filament could be determined. For each snapshot, the values of IQ signals (or amplitude and phase) were calculated to obtain a time dependency.

In addition, to provide a more complete analysis, the shape of the filament was changed in the performed simulations. This was conducted based on the experimental evidence that suggests that a simple circular filament model may not be accurate for all conditions [18]. The forms included strip filaments and filaments stretched in both the poloidal and radial directions. A range of their parameters were investigated and are presented in Table 4 and Table 5.

## 3. Circular Filaments

Modelling in the work in [15] was undertaken to answer the question how DBS signals of a given probing frequency in Globus-M would react to the presence of a circular filament in the proximity of its cut-off, which in that case was 48 GHz. However, this was not enough to explain all the data collected using the Globus-M/M2 DBS system, which lead to keen interest in how other channels (more specifically of higher frequencies with cut-offs in deeper regions, but the SOL region was also investigated) could be influenced by a circular filament in the periphery where they develop [4,5,6,17]. In this work, the calculations of DBS signals for various probing frequencies (Table 1) were performed. The filament was positioned near the separatrix at the cut-off radius of the 48 GHz probing frequency, while the signals at other radii were analyzed.

**Figure 4:**
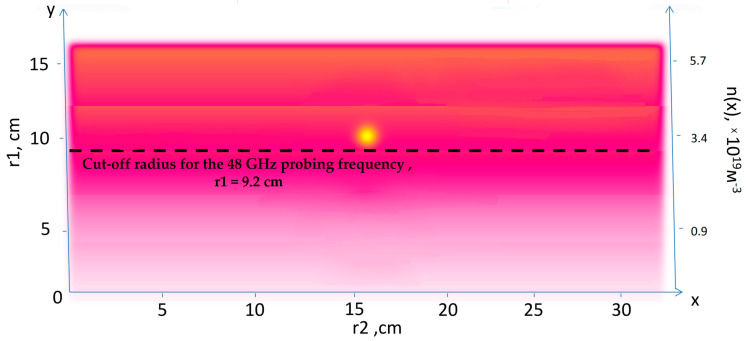
Density perturbations with an introduced circular filament (yellow circle).

Density perturbations with a circular filament near the cut-off of the 48 GHz probing frequency introduced on top of the bulk plasma density are presented in Figure 4. The radial position of the filament was not changed, but the filament moved with a constant velocity of 10 km/s in the poloidal direction (in the direction of the *x* axis).

**Figure 5:**
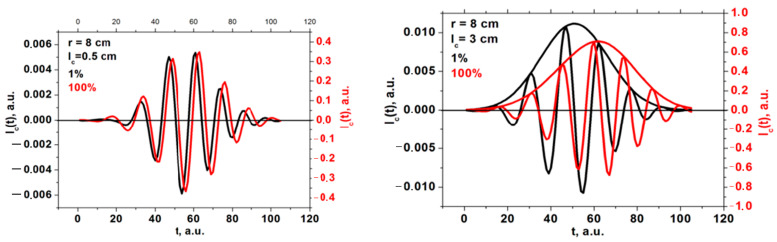
DBS signals for the 48 GHz probing frequency in the case of a circular filament of (**left**) 0.5 cm diameter and (**right**) 3 cm diameter. The black signals correspond to filaments with an amplitude of 1% of density at cutoff of the probing wave, and the red signals correspond to an amplitude of 100%.

For the conditions previously described, a variety of DBS signals were calculated, with several examples presented in Figure 5. They included two pairs of signals for the probing frequency of 48 GHz obtained for circular filaments of different diameters (left—0.5; right—3 cm) and amplitudes (black line—1%; red line—100%). After a certain critical size of the circular filament was reached, a significant delay in the formation of the filament between the low and high amplitude case was seen (right in Figure 5). This was not seen for the 0.5 cm filament and was not observed for any other intermediate diameter values.

**Figure 6:**
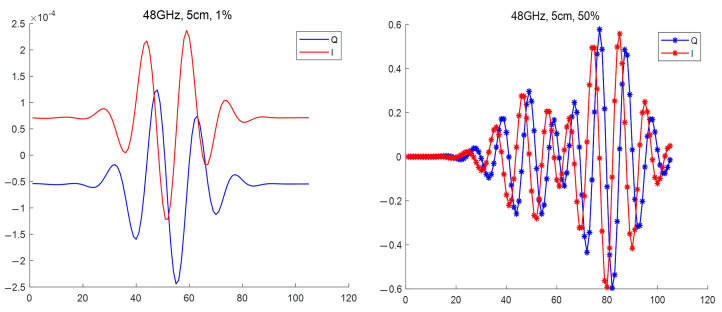
DBS IQ signals for the 48 GHz probing frequency for a circular filament with a 5 cm diameter: (**left**) amplitude of 1% of density at cutoff of the probing wave, (**right**) amplitude of 100% of density at cutoff of the probing wave.

Another example of the influence of the size of the circular filament on the DBS signals is presented in Figure 6. It contains the IQ signals obtained for a filament with a 5 cm diameter. The comparison of the low (1%) and high (50%) amplitude cases led to the observation that the signal frequency significantly increased after transitioning to a non-linear regime.

**Figure 7:**
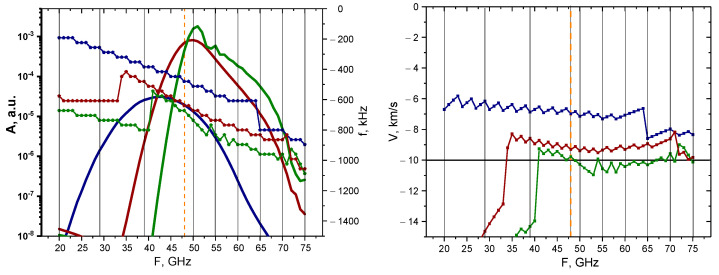
For the circular filament with an amplitude of 0.1%: (**left**) dependency of the DBS signal amplitude (bold lines with scale on the left) and frequency (lines with pentagon shapes with scale on the right) on the probing frequency; (**right**) dependency of the calculated velocity on the probing frequency. The navy-blue lines are the circular filament with a 3 cm diameter, the wine-red are that of 1.5 cm, and the olive-green are that of 0.5 cm. The black vertical lines indicate the probing frequencies available in Globus-M2, and the vertical orange dashed line indicates the position of the filament at cutoff of the 48 GHz probing wave. The horizontal line marks the set 10 km/s filament velocity.

A more systematic approach was then taken to determine how signals of different probing frequencies react to the presence of circular filaments of different sizes. The linear case was investigated with calculations undertaken for the 0.1% amplitude filament. The results of these calculations are presented in Figure 7. The dependency of the maximum of the signal amplitude, as well as its main frequency component, is demonstrated on the left with the bold lines representing the change in signal amplitude depending on the probing frequency (scale on the left), while the other three lines with pentagon shapes represent the signal frequency behavior (scale on the right). The colors correspond to different circular filament diameters, with navy-blue describing the 3 cm diameter filament, wine-red describing that of 1.5 cm, and olive-green describing that of 0.5 cm. The vertical lines indicate the probing frequencies installed on the DBS system in the Globus-M2 tokamak (i.e., 20, 29, 39, 48, 50, 55, 60, 65, 70, and 75 GHz). Additionally, the orange dashed vertical line highlights the position of the filament at the cut-off radius of the 48 GHz probing frequency. In the linear case (0.1% amplitude), we observed a steady decrease in the signal frequency, while the signal amplitude experienced a steady increase until a certain peak was reached, after which a gradual decrease followed. The maximum of the signal amplitude took place around the 48 GHz frequency, where the filament was located in the model. The values of the signal amplitude also suggested that the DBS diagnostic would only be able to detect the filaments using channels with a range of probing frequencies of 40–55 GHz.

The Doppler frequency shift (signal frequency) was then used to calculate the filament velocity by the formula V=Δω/k, where $k=2k_{0}\sin\alpha$ is the wave vector detected by the DBS method. The motion of the filament in the poloidal direction was set at 10 km/s (horizontal line on the right), which allowed us to meaningfully analyze the estimated velocities. In the case of the 0.5 cm diameter filament, the results were accurate for the 35–75 GHz probing frequencies, while the other two cases (blue line—3 cm and red line—1.5 cm) provided velocity measurements of higher values.

**Figure 8:**
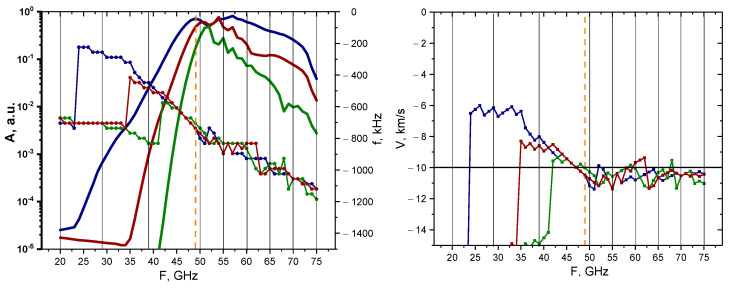
For the circular filament with an amplitude of 50%: (**left**) dependency of the DBS signal amplitude (bold lines with scale on the left) and frequency (lines with pentagon shapes with scale on the right) on the probing frequency; (**right**) dependency of the calculated velocity on the probing frequency. The navy-blue lines are the circular filament with a 3 cm diameter, the wine-red are that of 1.5 cm, and the olive-green are that of 0.5 cm. The black vertical lines indicate the probing frequencies available in Globus-M2, and the vertical orange dashed line indicates the position of the filament at cutoff of the 48 GHz probing wave. The horizontal line marks the set 10 km/s filament velocity.

An analysis was carried out for the non-linear case (50% filament amplitude), and the results of the calculations are presented in Figure 8. The behavior of the signal amplitudes and frequency remained similar to the linear case; however, the values of the signal amplitude implied that the filaments would be observable using a wider range of probing frequencies of 45–65 GHz. Additionally, filaments of all sizes exhibited very similar behaviors and values, but in the linear case, a greater discrepancy was observed between the 3 cm filament and the others. The velocity for all the filaments was slightly above the set 10 km/s value after 47 GHz, but before that the values were lower and differed.

### 3.1. Motion of the Filament in the Poloidal Direction from Different Radial Positions

**Figure 9:**
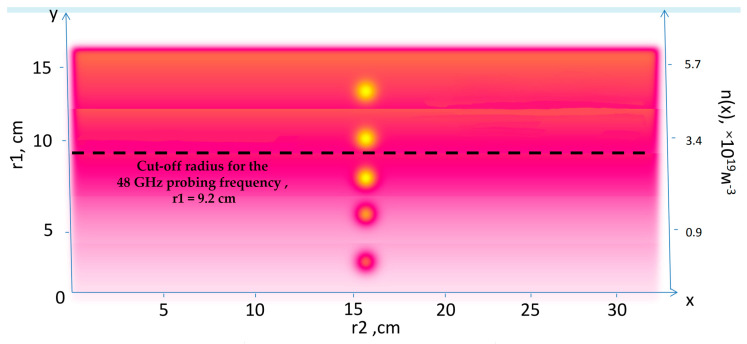
Density perturbations with an introduced series of circular filaments (yellow circles) positioned at different radii.

Apart from a fixed radial filament position, calculations were also performed for filaments positioned at different radii (Table 2). Figure 9 presents the density perturbations with a series of circular filaments positioned at different radii and introduced on top of the bulk plasma density. The circular filament moved in the poloidal direction (in the direction of the *x* axis) at a 10 km/s velocity.

**Figure 10:**
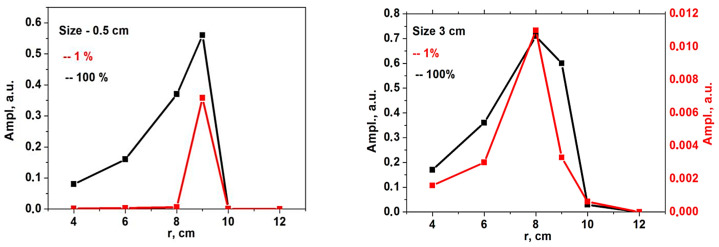
The dependency of the signal amplitude on the radial position of the circular filament of (**left**) 0.5 cm diameter and (**right**) 3 cm diameter. The red line corresponds to the filament with an amplitude of 1% of density at cutoff of the probing wave 48 GHz where the filament was positioned, and the black line corresponds to an amplitude of 100%.

To investigate the influence of the position of the filament, the DBS signals for a given probing frequency with the circular filament at different radii were calculated, and the signal amplitude was analyzed. The results for the 48 GHz probing frequency are presented in Figure 10. Filaments of different sizes (left—0.5 cm, right—3 cm) and for different amplitudes (red line—1%; black line—100%) were also compared. One may note that a good resolution was only observed in the linear case for the small filament. With increasing the filament size and amplitude, the spatial resolution dropped.

**Figure 11:**
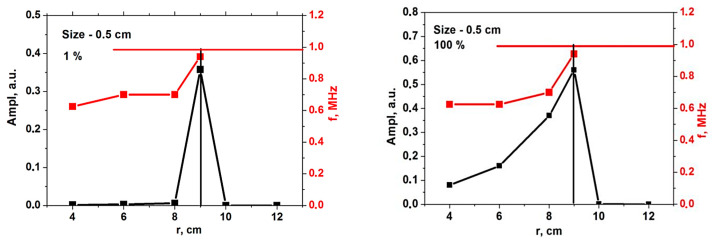
For the circular filament, the dependency of the signal amplitude (black line, scale on the left) and frequency (red line, scale on the right) on the radial position of the circular filament (**left**) with an amplitude of 1% of density at cutoff of the probing wave 48 GHz where the filament was positioned, and (**right**) a filament with an amplitude of 100%. The horizontal red line is the frequency predicted by the formula for the Doppler shift in the Born approximation.

The signal frequency was investigated for the 0.5 cm filament. It is presented in Figure 11 and is depicted by the red line. In both the linear and non-linear cases, the signal frequency coincided with the frequency predicted by the formula for the Doppler shift in the Born approximation (red horizontal line) only when the filament was positioned in the cutoff of the given frequency. For all other positions, the frequency values were lower.

### 3.2. Two-Dimensional Motion of the Filament

**Figure 12:**
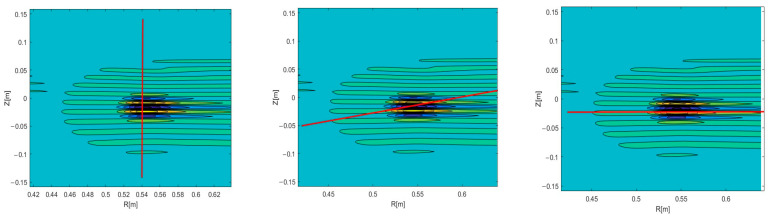
Two-dimensional DBS signal map for the circular filament positioned at the 48 GHz probing frequency. The red line indicates the trajectory: (**left**) poloidal motion, (**middle**) motion in both the poloidal and radial directions, (**right**) radial motion.

Experiments on various tokamaks demonstrated that filaments can travel both in the poloidal and the radial directions [17]. Complex trajectories were also observed, where the filaments had both velocity components. These observations made this an aspect of interest to model. In this work, we additionally investigated the possible effect that the radial velocity component has on DBS signals. The circular filament positioned at a radius 0.54 m of the 48 GHz probing frequency was investigated. Two-dimensional maps of the velocity signals were obtained, and the results are presented in Figure 12. The red lines indicate the analyzed trajectory. DBS signals were extracted at a chosen radius, which corresponded to a specific probing frequency.

**Figure 13:**
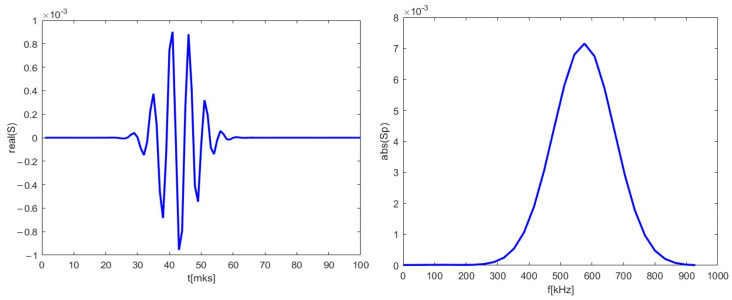
(**left**) DBS signal for the 48 GHz probing frequency; (**right**) calculated spectrum of the signal on the left.

The 48 GHz signal for poloidal motion (left in Figure 12) was investigated and presented in Figure 13. It was similar to the ones presented in previous sections and was used as reference for the signals calculated with added radial components. Its spectrum was calculated and is presented on the left. The main frequency component was 590 kHz.

**Figure 14:**
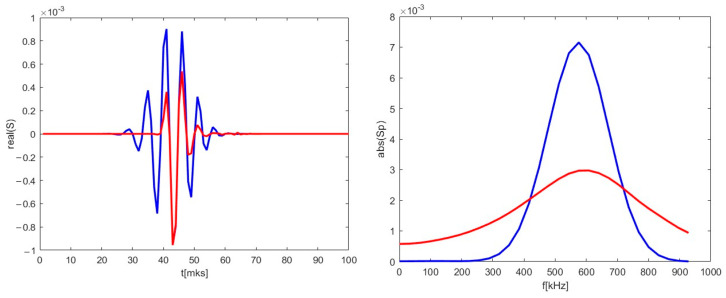
(**left**) DBS signals for the 48 GHz probing frequency; (**right**) calculated spectra of the signal on the left. The blue lines correspond to the reference signal from Figure 13. The red lines correspond to the calculations for trajectory in the middle in Figure 12.

A radial velocity component was introduced, and calculations were carried out for the new trajectory. As an example, the 2D map is presented in the middle in Figure 12. The new DBS signal is shown in the form of a red line in Figure 14. Along with that, the reference signal from Figure 13 is presented for comparison as a blue line. Some differences were observable for the new trajectory. The length of the new signal decreased significantly. In addition, while the largest peaks remained the same, the smaller ones decreased in amplitude and shifted closer toward the main ones. This was also highlighted using the calculated spectrum on the right. The main signal frequency component stayed at 590 kHz, but the spectrum widened in comparison to the poloidally moving filament.

**Figure 15:**
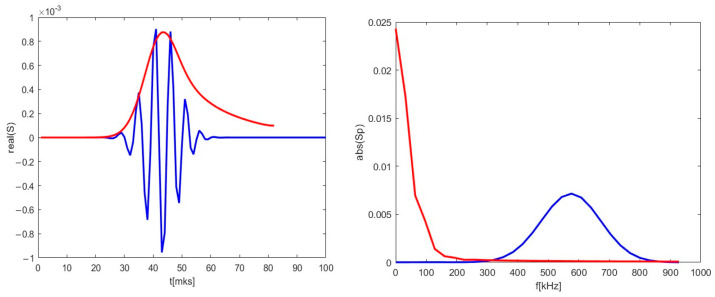
(**left**) DBS signals for the 48 GHz probing frequency; (**right**) calculated spectra of the signal on the left. The blue lines correspond to the reference signal from Figure 13. The red lines correspond to the calculations for trajectory in Figure 12.

The strictly radial motion presented on the right in Figure 12 was investigated. The obtained DBS signal is depicted in Figure 15 as a red line alongside the reference signal for comparison. For the case of the purely radial motion, the signal was always a single peak spanning over the whole signal length. The signal frequency was much lower than in the case of the circular filament moving poloidally, as the calculated spectrum on the right has its maximum at 1 kHz.

**Figure 16:**
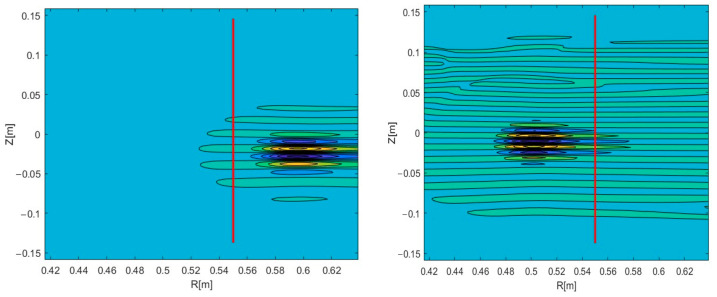
Two-dimensional DBS signal map for the circular filament positioned at the 48 GHz probing frequency: (**left**) 39 GHz, (**right**) 55 GHz probing frequency. The red line indicates the trajectory.

Signals of other frequencies were investigated while the filament was still positioned at the radius 0.54 m of the 48 GHz probing frequency so as to observe the influence of the radial component on the DBS signals. The strictly poloidal motion at a velocity of 10 km/s was studied. The 2D maps of the signals for the 39 GHz (left in Figure 16) and 55 GHz (right in Figure 16) were calculated.

**Figure 17:**
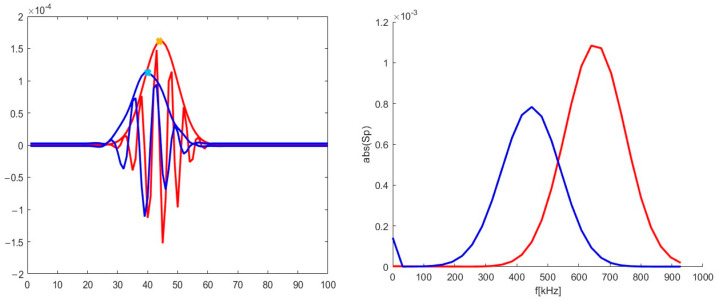
(**left**) DBS signals; (**right**) calculated spectra for the signal on the left. The blue lines correspond to the 39 GHz probing frequency, and the red lines correspond to 55 GHz.

The differences in the signals at various probing frequencies (or radii) were investigated and are presented in Figure 17. The blue line corresponds to the 39 GHz probing frequency, while the red one corresponds to 55 GHz. One may observe that the signals differed in amplitude and frequency in the spectra on the right. There was a shift of 3 ms between the two signals, with the 39 GHz signal forming earlier than the 55 GHz one. This shift cannot be explained by the motion in the radial direction, however there is data to explain this phenomenon. It is believed to be associated with the stretching and tilt of turbulence eddies [22].

**Figure 18:**
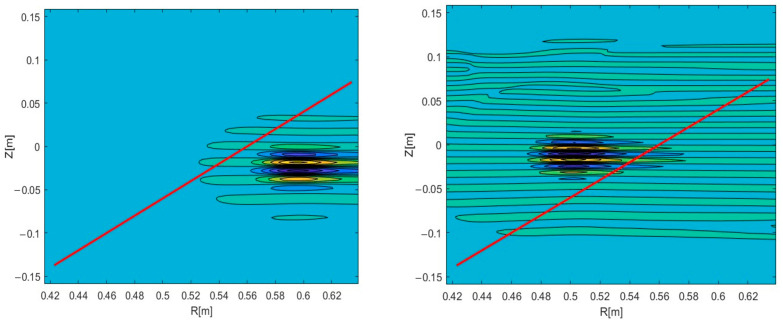
Two-dimensional DBS signal map for the circular filament positioned at the 48 GHz probing frequency: (**left**) 39 GHz; (**right**) 55 GHz probing frequency. The red line indicates the trajectory.

These parameters were also calculated for the case of an added radial velocity of 10 km/s with the circular filament moving in the direction of the increasing radius. The 2D maps of the DBS signals for the 39 GHz (left) and 55 GHz (right) probing frequencies are presented in Figure 18.

**Figure 19:**
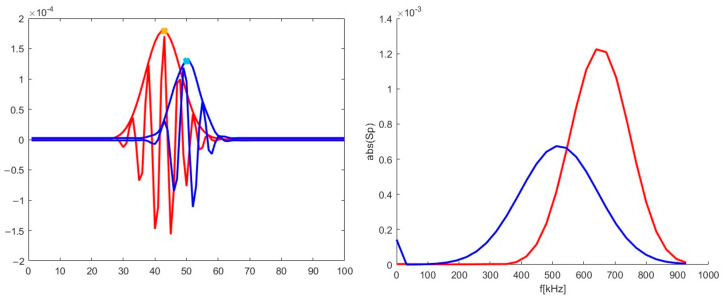
(**left**) DBS signals; (**right**) calculated spectra for the signal on the left. The blue lines correspond to the 39 GHz probing frequency, and the red line corresponds to 55 GHz.

The signals for the 39 and 55 GHz frequencies were extracted from the 2D maps in Figure 18. They are presented in Figure 19. Just as in the previous scenario, the amplitude of the 39 GHz signal was smaller than the 55 GHz one. In the spectrum on the right, one can see that the value of the 39 GHz signal frequency is now around 510 kHz rather than 450 kHz as was the case in the purely poloidal motion scenario. The shift between the two signals was also different with the radial motion introduced. The 55 GHz frequency signal now forms earlier than the 39 GHz one with a 7 ms delay. This is explained by the motion of the filament from the inner to the outer plasma regions. The 7 ms value was larger than the 3 ms delay between the signals in the case of the poloidal motion, meaning that the turbulence stretching and tilt cannot be playing the only key role in the formation of the DBS signal.

## 4. Strip Filaments

**Figure 20:**
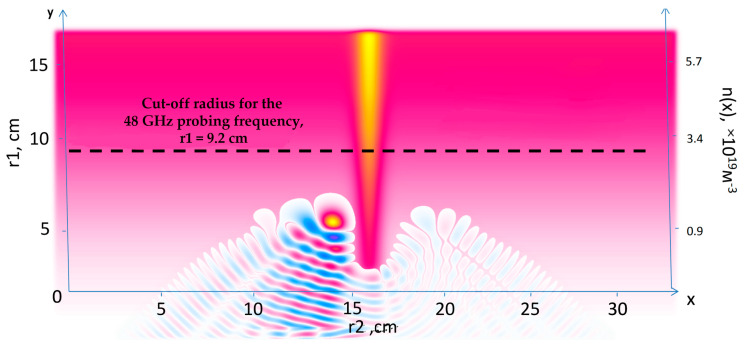
Density perturbations with an introduced strip filament (yellow vertical line). The electric field distribution is also presented.

The circular model for filaments was unable to explain several types of detected filaments. This raised questions as to what other models could be applied and investigated. For instance, there were observations of filaments radially localized in a large area with no delay between the detected filaments between DBS channels. This led to the idea of introducing filaments in the form of a strip stretching across the whole box where the modelling takes place. This ensured that the influence of the filament could be, to some extent, observed in a wide range of radii. Figure 20 presents the density perturbations with the strip model filament introduced on top of the bulk plasma density spanning the radial direction at a set poloidal coordinate. Additionally, the electric field distribution is presented.

**Figure 21:**
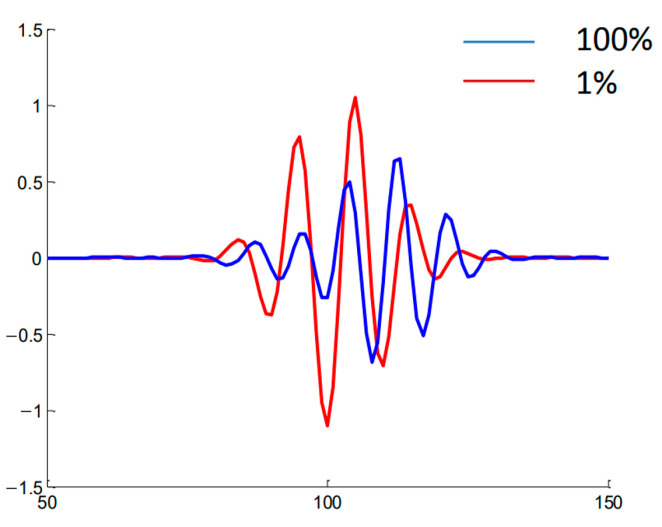
The DBS signal for the strip filament. Red line corresponds to the filament with an amplitude of 1% of density at cutoff of the probing wave 48 GHz where the filament was positioned, and the blue line corresponds to an amplitude of 100%.

DBS signals were calculated for the conditions, and the parameters are presented in Table 4. An example of the obtained signals is presented in Figure 21. Two cases were analyzed with the red line representing the linear one (1% filament amplitude), and the blue line—the non-linear case (100% filament amplitude). One may observe that there was a delay between the development of the filament in the non-linear regime in comparison to the linear one.

**Figure 22:**
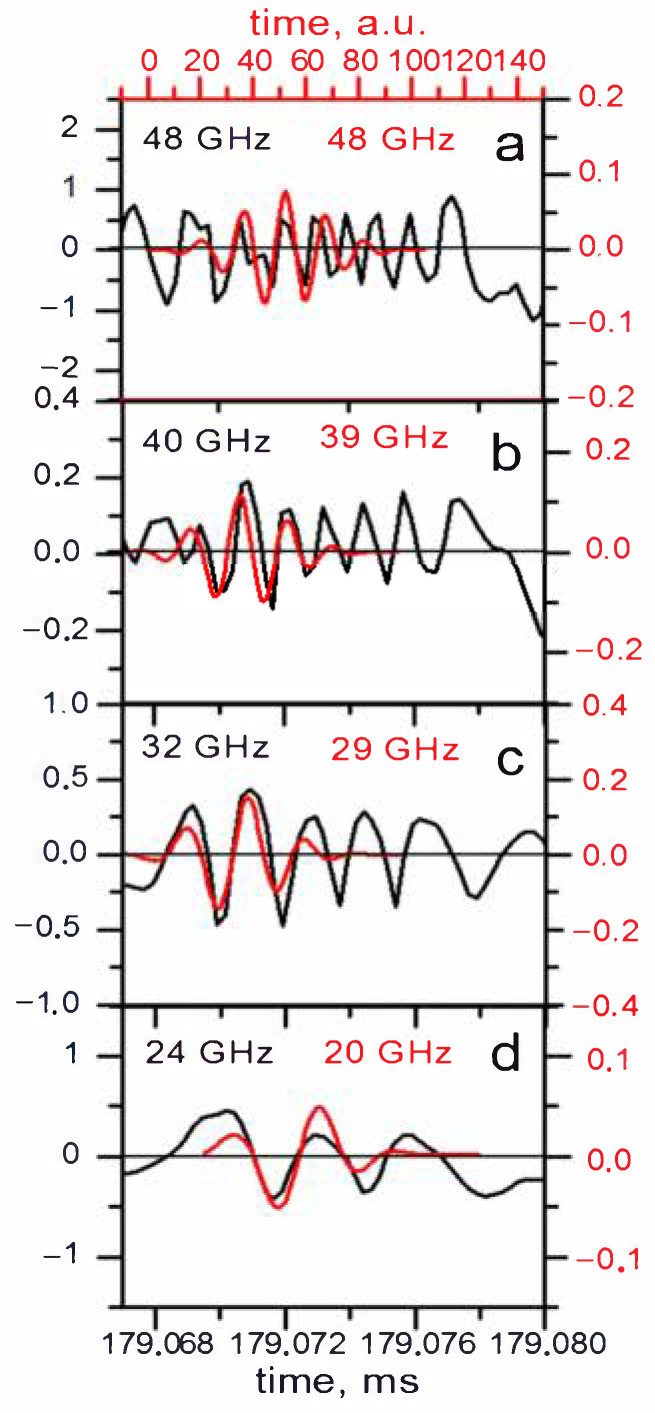
Experimental DBS signals (black lines) and calculated DBS signals for a strip filament model (red lines) for different probing frequencies.

This model was also compared to the filaments observed in Globus-M, and the results are presented in Figure 22. The calculated signal for the filament in the form of a strip with a width of 1.5 cm and amplitude of 1% is shown in red, while the black lines are the experimental data collected using DBS. The main issue with this model was the fact that the modelled signal was shorter than the signal in the experiment. Additionally, in the experiment, the frequency of the signal changed for different probing frequencies, while that did not take place in the calculations.

## 5. Stretched Filaments

**Figure 23:**
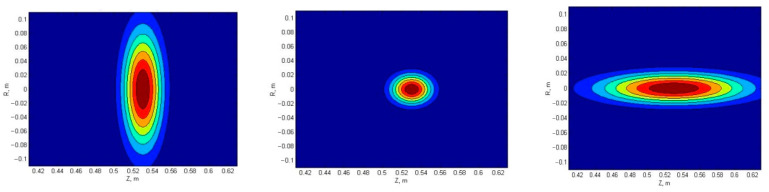
Filament density distribution: (**left**) radially stretched, (**middle**) circular, and (**right**) poloidally stretched filament.

Experiments on various tokamaks have highlighted that filaments of other shapes can form in plasma [18]. The gathered data showed that filaments can stretch in both the radial and poloidal directions. This has caused interest, as some filaments observed in Globus-M2 could not be explained by the circular and strip filament models. The filament density used for the performed simulations is presented in Figure 23. For comparison, the circular filament is shown in the middle, and the stretched filaments are shown on either side of it. For a given size of the filament, it was stretched with proportions of 1:2 or 1:4.

### 5.1. Radially Stretched Filaments

**Figure 24:**
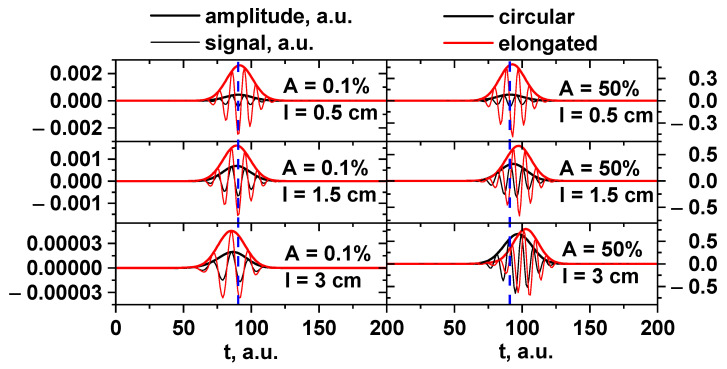
DBS signals (bold lines) and amplitude (regular lines) for the circular filament (black lines) and radially stretched filament (red lines): (**left**) filament amplitude of 0.1% of density at cutoff of the probing wave 48 GHz where the filament was positioned; (**right**) filament amplitude of 50%. Different filament diameters: (**top**) 0.5 cm, (**middle**) 1.5 cm, and (**bottom**) 3 cm.

The radially stretched filament with proportions of 1:2 was compared to the circular one. Differently sized filaments and amplitudes (linear and non-linear regimes) were investigated. The filaments were positioned at the cut-off radius of the 48 GHz probing frequency, and the signals for the 48 GHz frequency were obtained. The filament moved poloidally at a set velocity of 10 km/s. Figure 24 depicts the signals and amplitude for various conditions that were compared. The signal amplitude for the stretched filament was always greater. In the linear case (1% amplitude) there was no delay between the signals of the circular and stretched filament, while in the non-linear case (50% amplitude) a time offset was clearly visible, where the stretched filament signal developed later. The blue dashed vertical line can be used to observe this phenomenon.

**Figure 25:**
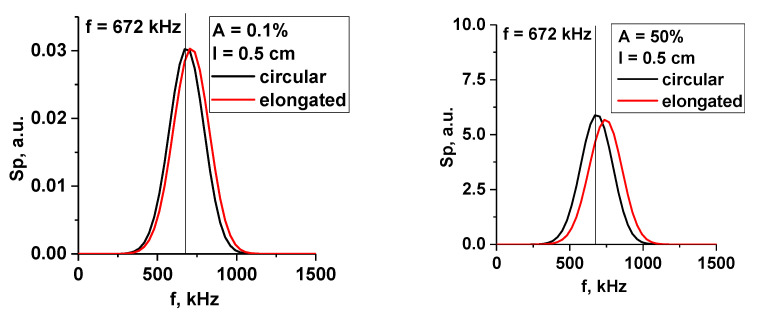
Spectrum of DBS signals for the 0.5 cm filament: (**left**) filament amplitude of 0.1% of density at cutoff of the probing wave 48 GHz where the filament was positioned; (**right**) amplitude of 50%. The black lines correspond to the circular filament, and the red lines correspond to the radially stretched filament.

The change in signal frequency was also of interest, so the spectra for the signals presented in Figure 24 were calculated. In Figure 25, the spectra of the filament with a 0.5 cm diameter for the linear (left) and non-linear (right) cases are presented. The circular filament frequency (black line) did not change with the transition from the linear to the non-linear regime, but the stretched filament frequency was always slightly larger and grew even more for the 50% amplitude.

**Figure 26:**
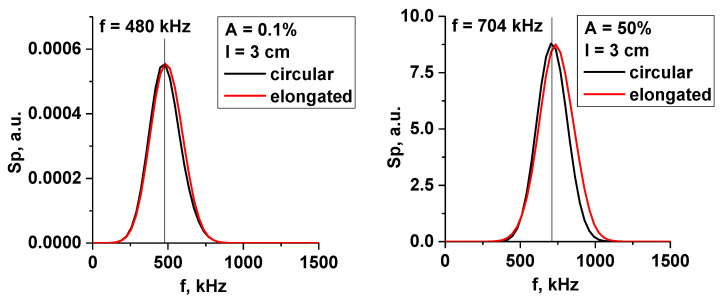
Spectrum of the DBS signals for the 3 cm filament: (**left**) filament amplitude of 0.1% of density at cutoff of the probing wave 48 GHz where the filament was positioned; (**right**) filament amplitude of 50%. The black lines correspond to the circular filament, and the red lines correspond to the radially stretched filament.

The largest filament with a 3 cm diameter is presented in Figure 26. The frequency in the linear case (0.1% amplitude) was smaller, with it being the same for the circular and stretched filaments. For the 50% amplitude case, the frequency was greater, with a value of 704 kHz, and its values were closer for the circular and stretched filaments. This was similar to the 1.5 cm filament.

**Figure 27:**
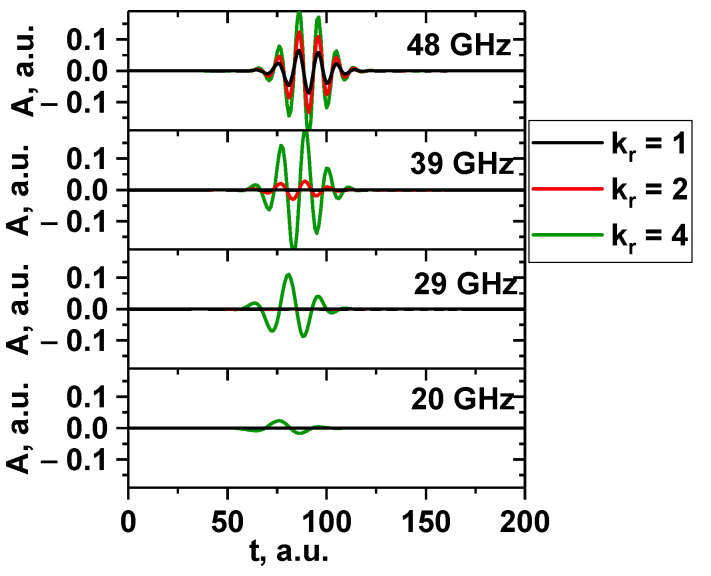
DBS signals for several probing frequencies. The circular (black line), slightly stretched with 1:2 proportions (red line), and very stretched with 1:4 proportions (green line) filaments are presented.

Signals for several probing frequencies were obtained and are presented in Figure 27. The filament was positioned at the cut-off radius for 48 GHz, and the signals for frequencies 48, 39, 29, and 20 GHz (DBS system in Globus-M/M2) were investigated. The filament moved poloidally at a set velocity of 10 km/s. The circular (black line), slightly stretched with 1:2 proportions (red line), and very stretched with 1:4 proportions (green line) filaments are presented. The diameter of the filament was 1.5 cm, and the amplitude was 1% of the density at cutoff of the probing wave (the linear case). The signals showed that, depending on the level of stretching of the filament, the number of signals that reacted to it changed. The more stretched the filament, the more channels formed peaks. This was similar to the strip model, where the signals on all channels were formed. The radially stretched filament could potentially be used to explain the experimentally observed filaments found on only two or three DBS signals.

**Figure 28:**
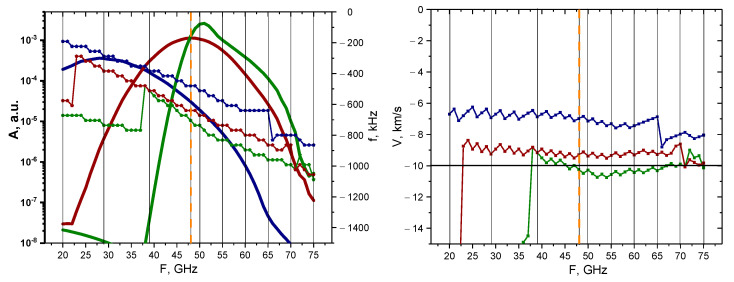
For the radially stretched filament with an amplitude of 0.1%: (**left**) dependency of the DBS signal amplitude (bold lines with scale on the left) and frequency (lines with pentagon shapes with scale on the right) on the probing frequency; (**right**) dependency of the calculated velocity on the probing frequency. The navy-blue lines are the circular filament with a 3 cm diameter, the wine-red are that of 1.5 cm, and the olive-green are that of 0.5 cm. The black vertical lines indicate the probing frequencies available in Globus-M2, and the vertical orange dashed line indicates the position of the filament at cutoff of the 48 GHz probing wave. The horizontal line marks the set 10 km/s filament velocity.

When analyzing the dependency of the signal amplitude, frequency, and velocity on the probing frequency in the linear case (0.1% amplitude) presented in Figure 28, the conclusion was that the radially stretched filament had the same dependencies, and even values, as the circular filament of the same size. The main difference was the signal amplitude for the 3 cm filament (blue line on the left). The amplitude values implied that it could potentially be detected by a lower range of probing frequencies of 20–45 GHz. The velocity on the right was very similar to the circular case, with the stretched filament with a diameter of 0.5 cm having the closest values to the set 10 km/s (olive-green line), and the others being lower in value.

**Figure 29:**
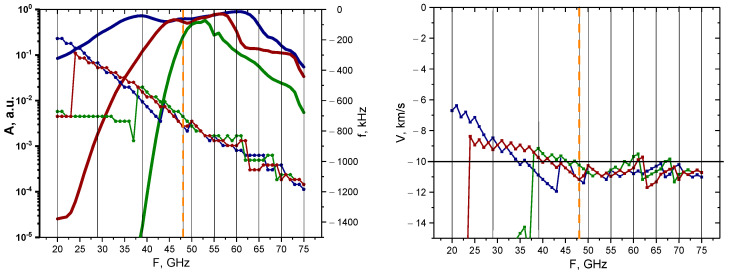
For the radially stretched filament with an amplitude of 50%: (**left**) dependency of the DBS signal amplitude (bold lines with scale on the left) and frequency (lines with pentagon shapes with scale on the right) on the probing frequency; (**right**) dependency of the calculated velocity on the probing frequency. The navy-blue lines are the circular filament with a 3 cm diameter, the wine-red are that of 1.5 cm, and the olive-green are that of 0.5 cm. The black vertical lines indicate the probing frequencies available in Globus-M2, and the vertical orange dashed line indicates the position of the filament at cutoff of the 48 GHz probing wave. The horizontal line marks the set 10 km/s filament velocity.

This remains true for the non-linear case with a 50% amplitude of the filament presented in Figure 29. The values of both the signal amplitude, frequency, and velocity were very similar in behavior for all the filament sizes (also observable for the circular filaments). The velocity vales for all the sizes were slightly larger than the expected 10 km/s after the 40 GHz frequency.

### 5.2. Poloidally Stretched Filaments

**Figure 30:**
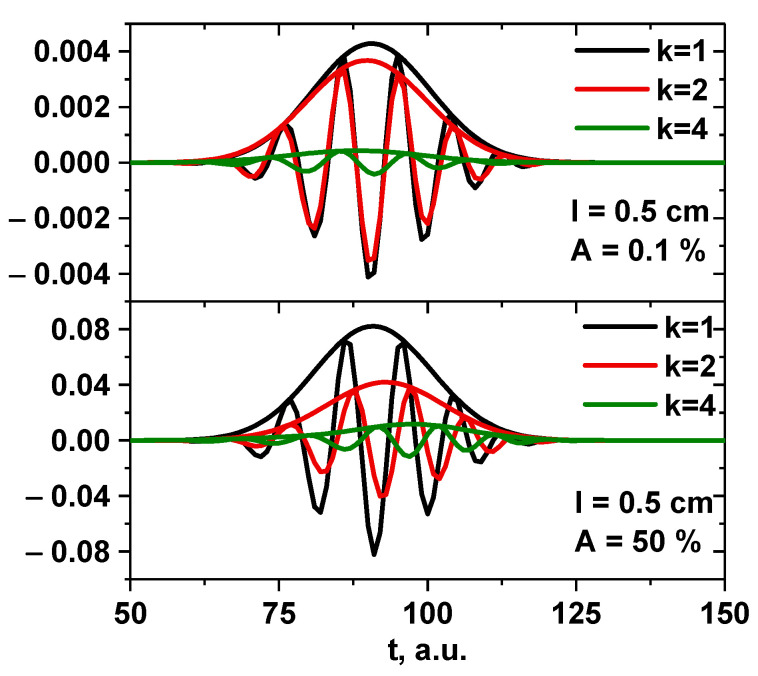
DBS signals for circular (black line), slightly poloidally stretched with 1:2 proportions (red line), and very poloidally stretched with 1:4 proportions (green line) filaments of 0.5 cm diameter: (**top**) amplitude of 0.1% of density at cutoff of the probing wave 48 GHz where the filament was positioned; (**bottom**) amplitude of 50%.

The poloidally stretched filaments with proportions of 1:2 and 1:4 were compared to the circular one. The DBS signals were calculated for the differently sized filaments and regimes. The filament was positioned at the cut-off radius for the 48 GHz probing frequency, and the signals for this frequency were obtained. It was set to move poloidally at a given velocity of 10 km/s. Figure 30 depicts the obtained signals. The signal amplitude for the poloidally stretched filament (red and green lines) decreased the more it was stretched compared to the circular filament (black line). It can also be said that while in the linear case (top) there was no delay between the signals, in the non-linear case (bottom) a time off-set was clearly visible where the stretched filament signal developed several ms later.

**Figure 31:**
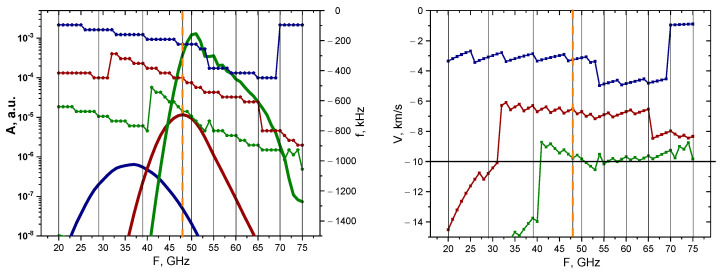
For the poloidally stretched filament with an amplitude of 0.1%: (**left**) dependency of the DBS signal amplitude (bold lines with scale on the left) and frequency (lines with pentagon shapes with scale on the right) on the probing frequency; (**right**) dependency of the calculated velocity on the probing frequency. The navy-blue lines are the circular filament with a 3 cm diameter, the wine-red are that of 1.5 cm, and the olive-green are that of 0.5 cm. The black vertical lines indicate the probing frequencies available in Globus-M2, and the vertical orange dashed line indicates the position of the filament at cutoff of the 48 GHz probing wave. The horizontal line marks the set 10 km/s filament velocity.

The behavior of the signal amplitude and Doppler frequency is presented in Figure 31. The poloidally stretched filament exhibited behavior that differed from the circular and radially stretched filaments of the same size. Judging by the calculated values of the amplitude of the 3 cm (bold blue line) and 1.5 cm (bold red line) filaments, one can come to the conclusion that no probing frequency signal would be able to detect them; however, for the 45–60 GHz frequencies, it was possible to observe the 0.5 cm poloidally stretched filament (bold green line). The frequencies and velocities were also calculated. Only the values for the 0.5 cm filament were close to the 10 km/s value after the 42 GHz probing frequency, with the 1.5 cm and 3 cm filaments having velocities significantly lower than the expected value.

**Figure 32:**
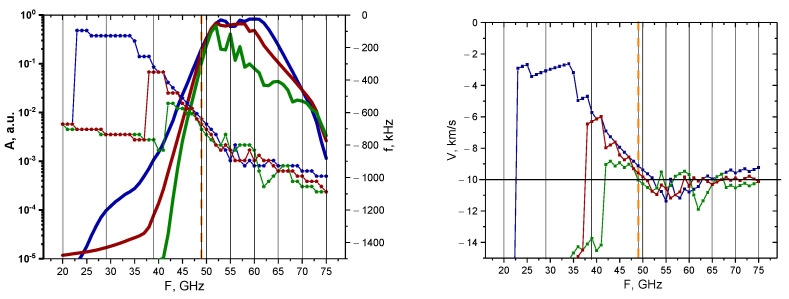
For the poloidally stretched filament with an amplitude of 50%: (**left**) dependency of the DBS signal amplitude (bold lines with scale on the left) and frequency (lines with pentagon shapes with scale on the right) on the probing frequency; (**right**) dependency of the calculated velocity on the probing frequency. The navy-blue lines are the circular filament with a 3 cm diameter, the wine-red are that of 1.5 cm, and the olive-green are that of 0.5 cm. The black vertical lines indicate the probing frequencies available in Globus-M2, and the vertical orange dashed line indicates the position of the filament at cutoff of the 48 GHz probing wave. The horizontal line marks the set 10 km/s filament velocity.

The non-linear case yielded different results, which are presented in Figure 32. The signal amplitude values were similar for all the probing frequencies, with the 45–70 GHz channels being able to detect the filament positioned at the 48 GHz cut-off radius. For the smaller probing frequencies, the Doppler shift values for the largest 3 cm filament differed from the 1.5 cm and 0.5 cm ones, which behaved similarly throughout. For probing frequencies below 48 GHz, the velocity had values that differed greatly from the anticipated 10 km/s; however, for channels deeper than 48 GHz, the values remained close to 10 km/s.

**Figure 33:**
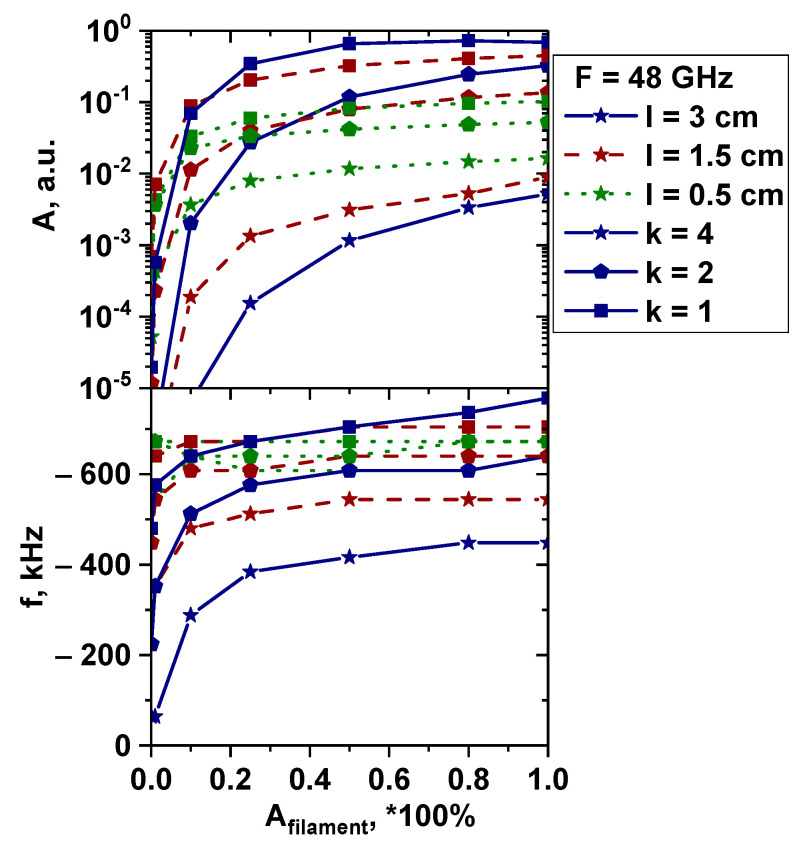
The dependency of the (**top**) signal amplitude and (**bottom**) signal frequency on filament amplitude. The blue lines correspond to 3 cm diameter filaments, the red lines correspond to 1.5 cm, and the green lines correspond to 0.5 cm. The lines with the squares correspond to the circular filament, the lines with pentagon shapes—poloidally stretched filament with proportions of 1:2, and the lines with stars—poloidally stretched filament with proportions of 1:4.

The transition from linear (lower than 50%) to non-linear (higher than 50%) was investigated for the poloidally stretched filament, as the DBS signals exhibited interesting characteristics that differed from all the previous cases. Figure 33 depicts the performed calculations and analysis for the 48 GHz probing frequency signal where the filament was positioned. The top figure shows the signal amplitude behavior, while the bottom one shows the signal frequency. The colors of the lines correspond to different filament sizes (blue line—3 cm diameter, red line—1.5 cm, and green line—0.5 cm), and the different symbols represent the degree of stretching (squares—circular filament, pentagon—stretched filament with proportions of 1:2, and stars—stretched filament with proportions of 1:4). In the linear case, the signal amplitude was larger the closer the filament size was to half the wavelength of the probing frequency near the cut-off position. This was in line with the Born approximation prediction. In the non-linear case, everything looked different. For the 1:2 poloidally stretched filament, as well as for the circular filament, the amplitude became larger the more its size increased and was similar in value, despite the poloidal size of the stretched filaments (6 cm) being much larger than half the wavelength. Only for the fourfold stretched filament did the tendency of the signal amplitude to decrease remain after its size changed from the optimal scattering size. The frequency in the linear case was inversely dependent on the size of the poloidal size of the filament; the smaller the length, the higher the frequency. Thus, the minimum frequency was achieved at the maximum size of the filament. This, together with the amplitude behavior of the signal, can be explained by the finite wavenumber resolution of the DBS diagnostics. So, in the presence of plasma fluctuations, the velocity determined using DBS will be significantly underestimated in relation to its true value only for filaments of large scale. In the non-linear case, there is a tendency to align all frequencies close to the frequencies that can be obtained in the linear case where the filament is appropriate in size. However, for very high elongation (1:4), the frequency remained too small even for very large amplitudes of filament. In addition, for the large circular filament, the frequency was generally overestimated.

## 6. Low-Density Profile

**Figure 34:**
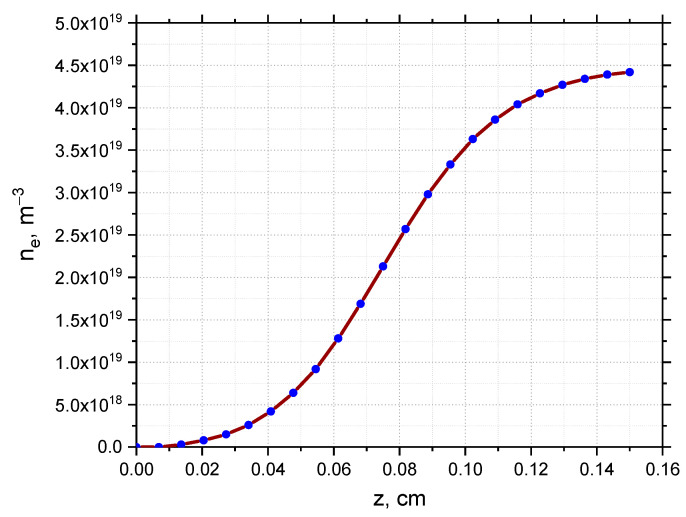
Experimental electron density profile for Globus-M2.

This section is devoted to the analysis of DBS signals for the conditions described in previous sections, but for a different electron density profile with density values lower than the one analyzed earlier. The experimental plasma density profile used for the computations is presented in Figure 34 and has values reaching n_e_ = 4.4 × 10^−19^ m^−3^ at y = 0.15 m (shot no. 36569 at Globus-M).

**Figure 35:**
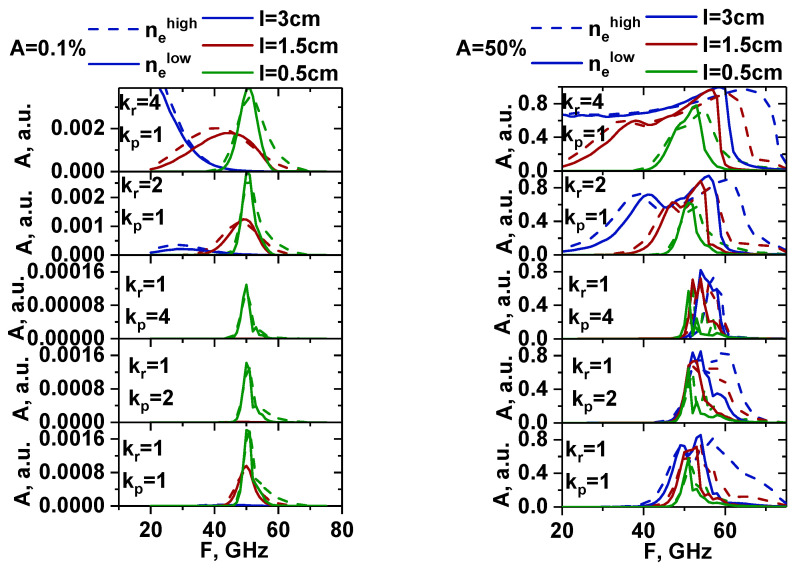
The dependency of the signal amplitude on different probing frequencies for the high-density profile from Figure 1 (dashed lines) and low-density profile from Figure 34 (regular lines): (**left**) filaments with an amplitude of 0.1% of density at cutoff of the probing wave 48 GHz where the filament was positioned; (**right**) filament with an amplitude of 50%. The blue lines correspond to 3 cm diameter filaments, the red lines correspond to 1.5 cm, and the green lines correspond to 0.5 cm. Different proportions of stretching of the filaments are presented in the figure.

The DBS signals were analyzed to see which channels of the reflectometer would be able to detect the forming filament. The frequency range remained the same, which led to the cut-off being shifted to deeper areas. It turned out that in both the linear (left in Figure 35) and non-linear cases (right in Figure 35), the filaments were visible for a smaller range of probing frequencies. This was indicated by the fact that the solid line profiles (low density profile) were more narrow than the dashed lines for all the filament sizes and forms. In the linear case, this was due to the cut-off positions shifting further away from the filament position compared to the high-density case. For the non-linear case, the observations can be explained by the same effect. All the characteristics of the filament discussed earlier were investigated, and no changes were revealed except for the localization shown.

## 7. Real Globus-M2 Geometry

Apart from the modelling performed in slab geometry, which was presented and discussed in detail in all the previous sections, the real Globus-M/M2 geometry was investigated. The question of its effects on the observable phenomena and DBS signals was raised, which is why additional simulations were performed to compare the two scenarios. The circular filament motion was along the magnetic field lines of the Globus-M/M2 tokamak at a constant velocity in the poloidal direction. To obtain this information, the experimental data of magnetic equilibrium (EFIT) and electron density (Thomson diagnostic) were used. A circular filament with a diameter of 3 cm and amplitudes of 1% (linear case) and 50% (non-linear case) was modelled. The filament was positioned at the cut-off of the 20 GHz probing frequency where the filament was generally observed.

**Figure 36:**
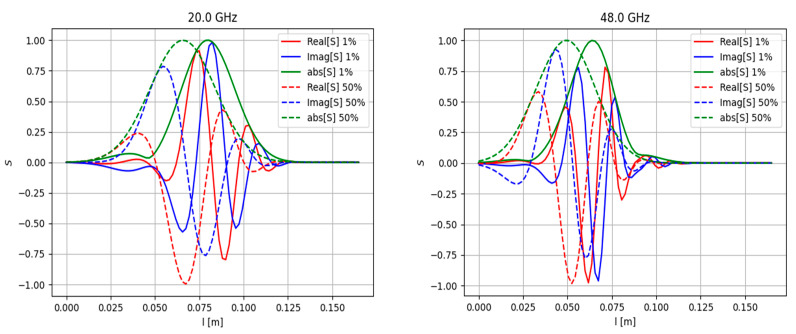
DBS signals calculated for the real Globus-M geometry (**left**) for the 20 GHz probing frequency and (**right**) for 48 GHz frequency. The regular lines correspond to filaments with amplitudes of 1% of density at cutoff of the probing wave, and the dashed lines correspond to filaments with amplitudes of 50% of density at cutoff of the probing wave.

The obtained DBS signals are presented in Figure 36. They were obtained for the 20 GHz frequency (left) where the filament was positioned and 48 GHz (right). For better comparison, the maximum signal amplitude was normalized. The signal for the filament with the higher amplitude of 50% developed earlier than the signal for the 1% filament. This was also the case for slab geometry simulations.

## 8. Conclusions

Two-dimensional full-wave simulations of backscattering off filaments were performed using the code IPF-FD3D to understand what kind of information about filaments can be extrapolated from signals of Doppler backscattering diagnostics. The DBS systems installed in Globus-M2 were analyzed, and different real experimental scenarios were examined. Computations were performed for a range of probing frequencies from 20 to 75 GHz of electromagnetic waves in O-mode

A circular filament was investigated in slab geometry. Different values of size, amplitude, position, and probing frequencies were analyzed. It was found that after a certain critical size of the circular filament was reached, a significant delay in the formation of the filament between the low and high amplitude case was seen. In addition, the signal frequency significantly increased after the transition to the non-linear regime. In the linear case, DBS was only able to detect the filaments using probing frequencies of 40–55 GHz, and the velocity values were accurate for 35–75 GHz frequencies for small filaments. For the non-linear case, the results indicated that the filaments were observable using a wider range of probing frequencies of 45–65 GHz, and the velocity for all the filament sizes was above the set value for deeper channels. In the linear and non-linear case, the signal frequency coincided with the frequency predicted by the formula for the Doppler shift in the Born approximation when the filament was positioned in the cut off of the given frequency, but it was lower as the position of the filament changed. The delay between different signals can be explained by the stretching and tilt of turbulence eddies or the introduction of the radial velocity.

Filaments in the form of a strip stretching across the whole box were modelled. There was a delay in the signals between the development of the filament in the non-linear regime in comparison to the linear one.

A radially stretched filament was investigated. In the linear case, there was no delay between the signals of the circular and stretched filaments, while in the non-linear case, a time offset was present, as the stretched filament signal developed later. It was found that the more stretched the filament, the more channels formed peaks. This was similar to the strip model. The radially stretched filament could potentially be used to explain the experimentally observed filaments found on only two or three DBS signals. In the linear and non-linear cases, the radially stretched filament had similar dependencies and values as the circular filament of the same size. The filament could potentially be detected by a lower range of probing frequencies of 20–45 GHz.

A poloidally stretched filament was investigated. In the linear case, there was no delay between the signals of the stretched and circular filaments, but in the non-linear case, a time off-set was presented, with the stretched filament signal developing several ms later. No probing frequency signal was able to detect large filaments; however, for the 45–60 GHz frequencies, it was possible to observe small filaments. Only the values for the small filament were close to the set value after the 42 GHz probing frequency, but others had significantly lower velocities. In the non-linear case, the 45–70 GHz channels were able to detect the filament positioned at the 48 GHz cut-off radius. For probing frequencies below 48 GHz, the velocity had values that differed greatly from the anticipated one; however, for channels deeper than 48 GHz, the values remained close to the set value.

For a low-density profile, in the linear and non-linear cases, the filaments were visible for a smaller range of probing frequencies. This was due to the cut-off positions shifting further away from the filament position compared to the high-density case.

The real Globus-M/M2 geometry was investigated. A circular filament moving along the magnetic lines in the poloidal direction was modelled. The signals in this case did not differ significantly from what was obtained in the slab geometry.

The next step in the study and modeling of the behavior of filaments in plasma includes more detailed calculations in the real geometry of the Globus-M2 tokamak to investigate the effect of magnetic field curvature on the resulting signals in the case of more complex filament forms and trajectories. Apart from that, when modelling filaments, background plasma turbulence will be introduced using the codes GENE and GKW. The effect of the tilting of filaments due to the presence of a shear will be studied. In addition, the results will be compared with DBS data collected during a specially designed experiment. Machine learning technologies will be applied to develop the process of recognizing filaments in experimental DBS signals.

## Figures and Tables

**Table 1:** Parameters of the performed modelling using the code IPF-FD3D.

Box size	40 × 24 cm = 1538 × 923 pts
Step size	dx = 1 pt = 0.026 cm
Equatorial plane	X = 10 cm = 384 pts
PML	Y = 0.625 cm = 24 pts
Plasma edge	Y = 2.08 cm
Antenna coordinates	X = 4 cm = 154, Y = 0
Antenna tilt angle	6 deg
Horn mouth	5.5 cm
Wave front	flat, Gaussian beam, w0 = 2.5 cm, z0 = 0

**Table 2:** Parameters of the performed modelling for circular filaments.

Frequencies	*20, 29, 39,* 48, *50, 55, 60, 65, 70, 75* GHz
Antenna tilt angle	13 degrees
Horn mouth	5.5
Wave front	flat
Diameter of the filament	0.5 cm, 1.5 cm, *3* cm
Amplitude of the filament	0.1%, 1%, 10%, *25%*, 50%, *80%*, 100%
Position of the filament	In the vicinity of the cut off 48 GHZ

**Table 3:** Parameters of the performed modelling for the motion of circular filaments.

Frequencies	48 GHz
Antenna tilt angle	13 degrees
Horn mouth	5.5
Wave front	flat
Diameter of the filament	0.5 cm, 1 cm, 1.5 cm, *5* cm
Amplitude of the filament	0.1%, 1%, 5%, 10%, 50%, *75%*, 100%
Position of the filament	4 cm, 6 cm, 8 cm, 10 cm, 12 cm

**Table 4:** Parameters of the performed modelling for the strip filament.

Frequencies	30, 35, 40, 45, 50, 55, 60, 65, 70, 75 GHz
Antenna tilt angle	13 deg
Horn mouth	5.5 cm
Wave front	flat
Diameter of the filament	0.5 cm, 1 cm, 1.5 cm, 3 cm, 5 cm, 6 cm
Amplitude of the filament	0.1%, 1%, 5%, 10%, 20%, 30%, 40%, 50%, 60%, 70%, 80%, 90%, 100%, 110%, 150%
Position of the filament	In the vicinity of the cut off

**Table 5:** Parameters of the performed modelling for the stretched filament.

Frequencies	20, 29, 39, 48, 50, 55, 60, 65, 70, 75 GHz
Antenna tilt angle	13 deg
Horn mouth	5.5 cm
Wave front	flat
Diameter of the filament	0.5 cm, 1.5 cm, 3 cm
Amplitude of the filament	0.1%, 1%, 10%, 25%, 50%, 80%, 100%
Position of the filament	In the vicinity of the cut off

## Data Availability

Not applicable.

## References

[1] Aymar R. (2002). ITER status, design and material objectives. J. Nucl. Mater..

[2] Linke J.M., Hirai T., Rödig M., Singheiser L.A. (2004). Performance of Plasma-Facing Materials Under Intense Thermal Loads in Tokamaks and Stellarators. Fusion Sci. Technol..

[3] D’Ippolito D.A., Myra J.R., Zweben S.J. (2011). Convective transport by intermittent blob-filaments: Comparison of theory and experiment. Phys. Plasmas.

[4] Fuchert G., Birkenmeier G., Carralero D., Lunt T., Manz P., Müller H.W., Nold B., Ramisch M., Rohde V., Stroth U. (2014). Blob properties in L- and H-mode from gas-puff imaging in ASDEX upgrade. Plasma Phys. Control. Fusion.

[5] Yun G.S., Lee W., Choi M.J., Lee J., Park H.K., Tobias B., Domier C.W., Luhmann N.C., Donné A.J.H., Team K. (2011). Two-Dimensional Visualization of Growth and Burst of the Edge-Localized Filaments in KSTAR H-Mode Plasmas. Phys. Rev. Lett..

[6] Spolaore M., Kovarík K., Stöckel J., Adamek J., Dejarnac R., Duran I., Komm M., Markovic T., Martines E., Panek R. (2017). Electromagnetic ELM and inter-ELM filaments detected in the COMPASS Scrape-Off Layer. Nucl. Mater. Energy.

[7] Zoletnik S., Anda G., Biedermann C., Carralero A.D., Cseh G., Dunai D., Killer C., Kocsis G., Krämer-Flecken A., Otte M. (2020). Multi-diagnostic analysis of plasma filaments in the island divertor. Plasma Phys. Control. Fusion.

[8] Conway G.D., Schirmer J., Klenge S., Suttrop W., Holzhauer E., ASDEX Upgrade Team (2004). Plasma rotation profile measurements using Doppler reflectometry. Plasma Phys. Control. Fusion.

[9] Bulanin V.V., Varfolomeev V.I., Gusev V.K., Ivanov A.E., Krikunov S.V., Kurskiev G.S., Larionov M.M., Minaev V.B., Patrov M.I., Petrov A.V. (2011). Observation of filaments on the Globus-M tokamak by Doppler reflectometry. Tech. Phys. Lett..

[10] Bulanin V.V., Gusev V.K., Khromov N.A., Kurskiev G.S., Minaev V.B., Patrov M.I., Petrov A.V., Petrov M.A., Petrov Y.V., Prisiazhniuk D. (2019). The study of filaments by the Doppler backscattering method in the 'Globus-M' tokamak. Nucl. Fusion.

[11] Yashin A.Y., Bulanin V.V., Petrov A.V., Gusev V.K., Kurskiev G.S., Minaev V.B., Patrov M.I., Petrov Y.V. (2019). Recent Doppler backscattering applications in Globus-M tokamak. JINST.

[12] Bulanin V.V., Gusev V.K., Kurskiev G.S., Minaev V.B., Patrov M.I., Petrov A.V., Petrov Y.V., Prisyazhnyuk D.V., Sakharov N.V., Solokha V.V. (2019). The Effect of Low-Frequency Magnetohydrodynamic Modes on the Development of Filaments in the Globus-M Tokamak. Tech. Phys. Lett..

[13] Yashin A., Bulanin V., Petrov A., Ponomarenko A. (2021). Review of Advanced Implementation of Doppler Backscattering Method in Globus-M. Appl. Sci..

[14] Trier E., Hennequin P., Pinzón J.R., Hoelzl M., Conway G.D., Happel T., Harrer G.F., Mink F., Orain F., Wolfrum E. Studying ELM filaments with Doppler reflectometry in ASDEX Upgrade. Proceedings of the 45th EPS Conference on Plasma Physics.

[15] Bulanin V.V., Gusakov E.Z., Gusev V.K., Zadvitskiy G., Lechte C., Heuraux S., Minaev V.B., Petrov A.V., Petrov Y.V., Sakharov N.V. (2020). Full-Wave Modeling of Doppler Backscattering from Filaments. Plasma Phys. Rep..

[16] Petrov Y.V., Gusev V.K., Sakharov N.V., Minaev V.B., Varfolomeev V.I., Dyachenko V.V., Balachenkov I.M., Bakharev N.N., Bondarchuk E.N., Bulanin V.V. (2022). Overview of GLOBUS-M2 spherical tokamak results at the enhanced values of magnetic field and plasma current. Nucl. Fusion.

[17] Lechte C. (2009). Investigation of the Scattering Efficiency in Doppler Reflectometry by Two-Dimensional Full-Wave Simulations. IEEE Trans. Plasma Sci..

[18] Kirk A., Counsell G.F., Cunningham G., Dowling J., Dunstan M., Meyer H., Price M., Saarelma S., Scannell R., Walsh M. (2007). Evolution of the pedestal on MAST and the implications for ELM power loadings. Plasma Phys. Control. Fusion.

[19] Ben Ayed N., Kirk A., Dudson B., Tallents S., Vann R.G.L., Wilson H.R., the MAST team (2009). Inter-ELM filaments and turbulent transport in the Mega-Amp Spherical Tokamak. Plasma Phys. Control. Fusion.

[20] Bulanin V.V., Yashin A.Y., Petrov A.V., Gusev V.K., Minaev V.B., Patrov M.I., Petrov Y.V., Prisiazhniuk D.V., Varfolomeev V.I. (2021). Doppler backscattering diagnostic with dual homodyne detection on the Globus-M compact spherical tokamak. Rev. Sci. Instrum..

[21] Yashin A.Y., Bulanin V.V., Gusev V.K., Minaev V.B., Petrov A.V., Petrov Y.V., Ponomarenko A.M., Varfolomeev V.I. (2022). Doppler backscattering systems on the Globus-M2 tokamak. JINST.

[22] Pinzón J.R., Estrada T., Happel T., Hennequin P., Blanco E., Stroth U., the ASDEX Upgrade and TJ-II Teams (2019). Measurement of the tilt angle of turbulent structures in magnetically confined plasmas using Doppler reflectometry. Plasma Phys. Control. Fusion.

